# Dispersal and habitat preference of juvenile emperor penguins—implications for conservation management

**DOI:** 10.1098/rsos.231800

**Published:** 2025-05-28

**Authors:** Azwianewi Benedict Makhado, Robert J M Crawford, Bruce M Dyer, Makhudu Masotla, Andrew Lowther

**Affiliations:** ^1^ Oceans and Coasts, Republic of South Africa Department of Forestry, Fisheries and the Environment, Cape Town, Western Cape, South Africa; ^2^ FitzPatrick Institute of African Ornithology, DSI-NRF Centre of Excellence, University of Cape Town, Rondebosch, South Africa; ^3^ Shanghai Ocean University College of Marine Living Resource Sciences and Management, Shanghai, People’s Republic of China; ^4^ Oceans and Coasts, Republic of South Africa Department of Forestry, Fisheries and the Environment, Cape Town, Western Cape, South Africa; ^5^ Research Department, Norwegian Polar Institute, Tromsø, Norway

**Keywords:** emperor penguins, dispersal, marginal ice zone, juvenile, Commission for the Conservation of Antarctic Marine Living Resources (CCAMLR), marine protected areas (MPAs)

## Abstract

Seabirds can disperse widely when searching for prey, particularly during nonbreeding periods. Conservation measures predominately focus on protecting breeding colonies, while spatial protection at sea is often based on knowledge of the distribution of breeding adults, despite accumulating evidence that marine habitats used by immature birds sometimes differ from those of adults. Juvenile emperor penguins from Atka Bay, west Dronning Maud Land, Antarctica, tracked immediately after fledging performed long migrations to the northern extents of the Convention for the Conservation of Antarctic Marine Living Resources subareas 48.4 and 48.6. Individuals did not remain long at their northern positions, before commencing a rapid southerly movement to within a few hundred km of the marginal ice zone (MIZ). The initial migratory movement was broadly synchronous across individuals. The southward movement and subsequent change to area-restricted searching were consistent with the MIZ representing a potentially important feeding habitat for juvenile emperor penguins. Spatio-temporal management mechanisms may be beneficial in reducing threats to these young penguins.

## 1. Introduction

Integrated knowledge of the spatial distribution of long-lived animals, including all life-history stages, is important to fully understand their movements and susceptibility to present and future spatial threats, and to inform appropriate conservation measures (CMs) [1]. However, most studies on the foraging ecology of marine vertebrates are restricted to breeding adults, although other life-history stages may account for half of the total population of long-lived animals [1]. For emperor penguins *Aptenodytes forsteri*, little is known about juvenile dispersal, a period when individuals may be vulnerable to increased mortality owing to their naive foraging behaviour [2–4].

The emperor penguin is an Antarctic circumpolar, sea-ice obligate species for most of its life [3] and is the only vertebrate species that breeds during the Antarctic winter [5]. From the beginning of their breeding season in April until the fledging of chicks in January, adults rely on the stability of fast sea ice on which they lay eggs and rear chicks [6]. Adults spend much of their time foraging within the pack ice in the Convention for the Conservation of Antarctic Marine Living Resources (CCAMLR) Convention Area, both during the breeding season [5,7] and post-breeding [3,8,9]. They are confined to waters that are covered at least seasonally by sea ice, whereas juveniles exploit more distant habitats when dispersing from colonies and have travelled more than 2400 km from their natal colony, mainly in open water [5,10–12]. A recent study at Atka Bay (70°37′ S, 08°09′ W) showed that juvenile emperor penguins travelled north of 50° S (the lowest recorded latitude was 48.37° S), which is 600 km further north than previously recorded. They travelled both beyond the boundaries of existing and planned conservation and management areas within the CCAMLR and outside the CCAMLR region. Therefore, as also noted by [2], present spatial management in the Southern Ocean may be insufficient to protect emperor penguins.

In 2009, the global population of emperor penguins was estimated at *ca* 238 000 breeding pairs, of which approximately 78 000 pairs (about 30% of the total) bred at 15 colonies in the Weddell Sea [6,13]. In 2020, the species numbered 256 500 pairs and was listed as ‘near threatened’ because it was anticipated that thickness duration of the fast ice and persistence of pack ice around Antarctica would be affected by climate change [6,14,15], including warming and factors that influence winds and other ocean properties [16–19]. Additionally, emperor penguins are subject to potentially increased competition with fisheries for prey, exposure to pollution and human disturbance [20]. In 2022, for similar reasons, the emperor penguin was listed as endangered under the USA’s Endangered Species Act [21].

In 2023, the establishment of several additional protection zones in the Ross Sea, East Antarctica, Antarctic Peninsula and Weddell Sea was considered by CCAMLR based on the known habitats and distributions of key species, including emperor penguins [2]. This was thought particularly important for conservation efforts in the Southern Ocean that target highly mobile species and age groups. However, fledgling emperor penguins travel extraordinary distances, and their wide distribution raises the question of how best to protect them from potential depletion of food resources, as exceptionally large marine protected areas (MPAs) would be required to cover the range of the species. Details of the operation of the proposed Weddell Sea MPA (WSMPA), should it be implemented, are still to be worked out.

Our study used telemetry data collected on juvenile emperor penguins from the Atka Bay colony to provide more information on their post-fledgling dispersal. It corroborated the findings of [2] that current conservation efforts in the Southern Ocean are insufficient to ensure the protection of these highly mobile penguins and that other spatial measures should be considered. However, we extended our analysis beyond general movement patterns to discern what probably constituted critical feeding habitat for juveniles during their first trip to sea. We then characterized the threats from human activities based on these habitat needs and outlined the current and proposed management solutions under discussion by international management bodies that may help address these threats. Finally, we proposed a path towards an evidence-based management strategy that responds to the dynamic nature of the habitat used by juvenile emperor penguins.

## 2. Material and methods

**Figure 1.**
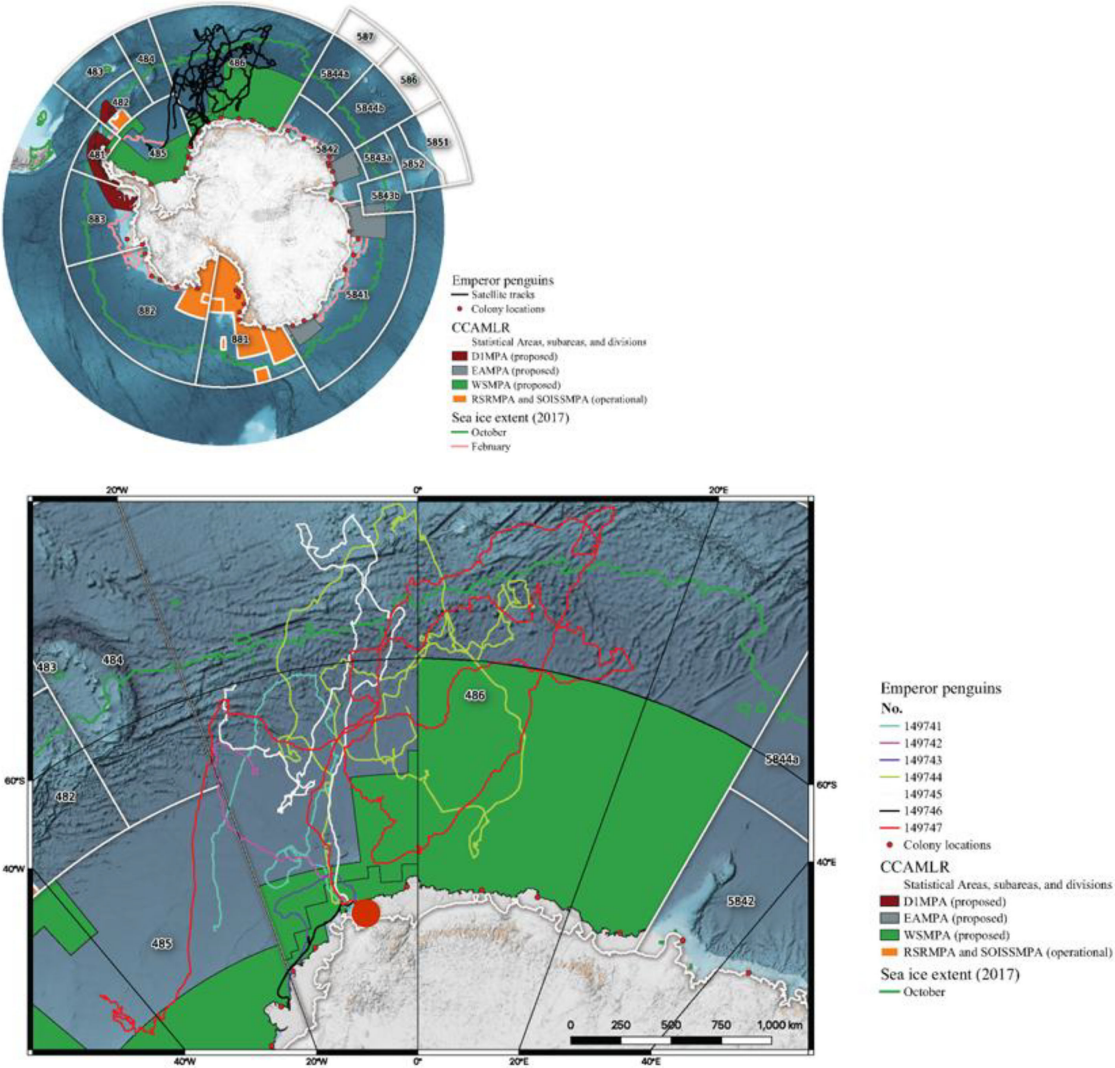
The CCAMLR Convention Area is subdivided into management areas using the designations of the Food and Agriculture Organization, with the fishery for Antarctic krill constrained to area 48, and area 58 subdivisions 4.1 and 4.2. Regional Fisheries Bodies border the Convention Area to its north (inset; numerically labelled white boxes). There are two designated marine protected areas within the Convention Area: the Ross Sea region MPA (RSRMPA, orange) and the South Orkney Island Southern Shelf MPA (not shown). There are also three MPAs under consideration by CCAMLR: the Domain 1 MPA (D1MPA), East Antarctic MPA (EAMPA) and Weddell Sea MPA (WSMPA). Sea ice extends north of almost all the MPAs during the winter (green line). Emperor penguin breeding colonies have a circumpolar distribution (red dots). The Atka Bay colony (main figure, large red circle) where the study was conducted is situated within the boundaries of the WSMPA, with instrumented juvenile penguins travelling over a wide area throughout subareas 48.5 and 48.6 during their first trip to sea (coloured lines represent individual penguin telemetry tracks). The figure was created using Quantarctica 3.16 (https://npolar.no/quantarctica/).

The study was conducted at the Atka Bay emperor penguin breeding colony (70°37′ S, 08°09′ W) in western Dronning Maud Land, Antarctica (figure 1) during the austral summer of 2016/2017. In the latter part of December, nine recently fledged juvenile emperor penguins were fitted with platform transmitter terminals (PTTs, 30 g Pico100, Microwave Telemetry, Columbia, MD; 35 g SPOT4, Wildlife Computers, Redmond, WA) programmed to transmit position signals for approximately eight months to the Argos satellite system (table 1). Birds were approached slowly and, when close enough, a shepherd’s hook was gently put around a penguin’s neck before it was captured. In order to minimize handling time and stress the chicks were not weighed. The instruments were attached using Tessa tape and Loctite 401 Super-glue, with the attachment fortified using cable ties. During instrument attachment, a bag was placed over the head of a fledgling to calm it. Each bird was carefully restrained by two people and released as soon as the glue had dried (<1 min). We selected chicks that had initiated their moult of down feathers and, as visually gauged, were in good condition, anticipating that well-nourished birds would have a better chance of survival than leaner individuals.

**Table 1.** Seven of the nine platform terminal transmitters applied to juvenile emperor penguins at the Atka Bay breeding colony successfully transmitted location data for longer than five days. (Three instruments transmitted for longer than six months, with one (149747) sending locations for almost 18 months.)

PTT ID	deployment	last transmission	duration (d)	positions
149741	4 January 2017	15 April 2017	100.6	8939
149742	4 January 2017	17 February 2017	43.5	4829
149743	5 January 2017	2 February 2017	27.5	3271
149744	30 December 2016	15 November 2017	323.3	8125
149745	30 December 2016	14 July 2017	195.5	11856
149746	30 December 2016	9 February 2017	40.3	615
149747	1 January 2017	26 May 2018	506.4	29144^a^

^a^ Given the possibility that this instrument continued to transmit after being moulted off, we note that in its first year (365 days) it provided 17454 positions).

### 2.1. Data processing and analysis

#### 2.1.1. Telemetry data

Raw PTT data were downloaded from the Argos-CLS data portal and imported into R statistical software framework (R version 4.2.2). To account for the temporal gaps and spatial errors that typify Argos location estimates for marine predators [22], telemetry data for each individual were filtered with a continuous time-correlated random walk model in the foieGras R package [16–19] using a regularized timestep of 8 h, which represented the average time gap between consecutive real location estimates. We estimated behavioural variation of individuals along their trajectory by applying a movement persistence model (mpm) from the foieGras R package to each trajectory separately [23]. The mpm provided a relative index between 0 (completely stationary) and 1 (straight line movement at relatively high speed) of how an animal moved along its trajectory, with lower values assumed to represent searching for food and higher values transitory movement [24].

#### 2.1.2. Sea ice covariate data

Circum-Antarctic, 6.25 km resolution daily ice maps based on microwave-measured data collected between 1 January 2017 and 31 December 2021 were downloaded from the University of Bremen repository (https://seaice.uni-bremen.de/data/amsr2/). We used a sea ice concentration of 15% as being representative of the northern edge of the pack ice zone (the start of the marginal ice zone, MIZ) [25], acknowledging that there is no universally accepted definition of this important environmental feature and that post-processing of sea ice data has uncertainty around the estimates of ice concentration. We note that the original description of the MIZ included the region where ice melt reduced salinity and led to chlorophyll blooms that extended up to 50 km from the ice edge [26]. However, the penguins we tracked would have met MIZ in April or May, on its northward advance, when ice may not have melted. For each penguin, a straight-line distance (km) was calculated between each location estimate and the nearest location at the same longitude of its temporally matched MIZ contour to provide an estimate of how far from the MIZ each juvenile penguin was while at sea. To represent how individual juvenile emperor penguins changed their movement behaviour, relative to how close they were to the MIZ, we then fitted a generalized linear mixed model (R package nlme) [27] using mpm as a response and the distance to the MIZ as a fixed predictor, individual as a random effect and a corCAR1() structure to account for temporal autocorrelation of the location estimates.

#### 2.1.3. Antarctic marine spatial management and winter sea ice coverage

Juvenile emperor penguins enter the water in late summer and spend most of autumn north of the ice zone, returning to it in late autumn and winter (e.g. [4]). To quantify how much of the MIZ falls within the boundaries of the proposed WSMPA [28], we calculated the daily proportion of coverage by sea ice over the 5-year period 2017−2021. We then used the dates when fledglings approached the MIZ to ascertain their approximate period of time within the WSMPA. We also similarly calculated the proportions of the East Antarctica MPA (EAMPA) and the Ross Sea Region MPA (RSRMPA) [28] covered by sea ice.

## 3. Results

Of the nine instruments deployed on fledglings, two transmitted for less than 5 days and were removed from further analysis. The remaining seven were active for 27–506 d (172.1 ± 176.2 d), providing a mean of 68.5 (± 3.9) raw locations per day (table 1). Only one instrument transmitted for more than 323 days. Had that instrument been moulted off at 365 days and subsequently drifted, the number of attached days would have varied from 27 to 365 d (157 ± 141 d). The instruments weighed 30−35 g and we expected they would sink if moulted off at sea. However, if they moulted onto, or became detached (e.g. [29]), while a bird was on, an iceberg, they could have drifted for some time. The juvenile penguins travelled as far north as 52° S and spanned 63° of longitude (figure 1).

**Figure 2.**
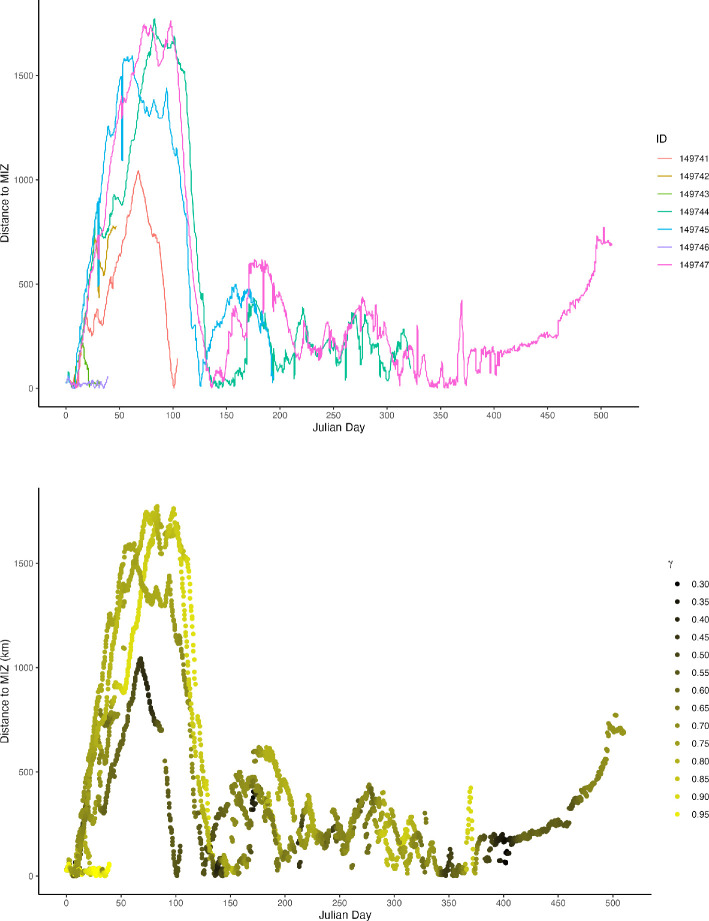
Instrumented juvenile emperor penguins initially travelled north from the Atka Bay colony until turning southwards to meet the marginal ice zone (MIZ) during April-May (top panel). Movement persistence estimated from modelled tracking data suggested that the initial movement north was directed, with individuals switching to a slower, less-persistence movement pattern once they returned south to the MIZ, consistent with assumed foraging behaviour (bottom panel).

Generally, all individuals moved consistently northwards to more than 1800 km north of Atka Bay (figure 1) and began returning southwards between mid-April and mid-May. This initial return was to the MIZ and then the two birds whose PTTs were still transmitting mostly remained within 500 km of the MIZ until mid-November (figure 2). As birds travelled towards the MIZ, there was a concomitant shift in their movement behaviour, with a significant reduction in how persistent their speed and direction of travel became (*t*_MIZ_ = −2.37, *p* = 0.02; figure 2).

**Figure 3.**
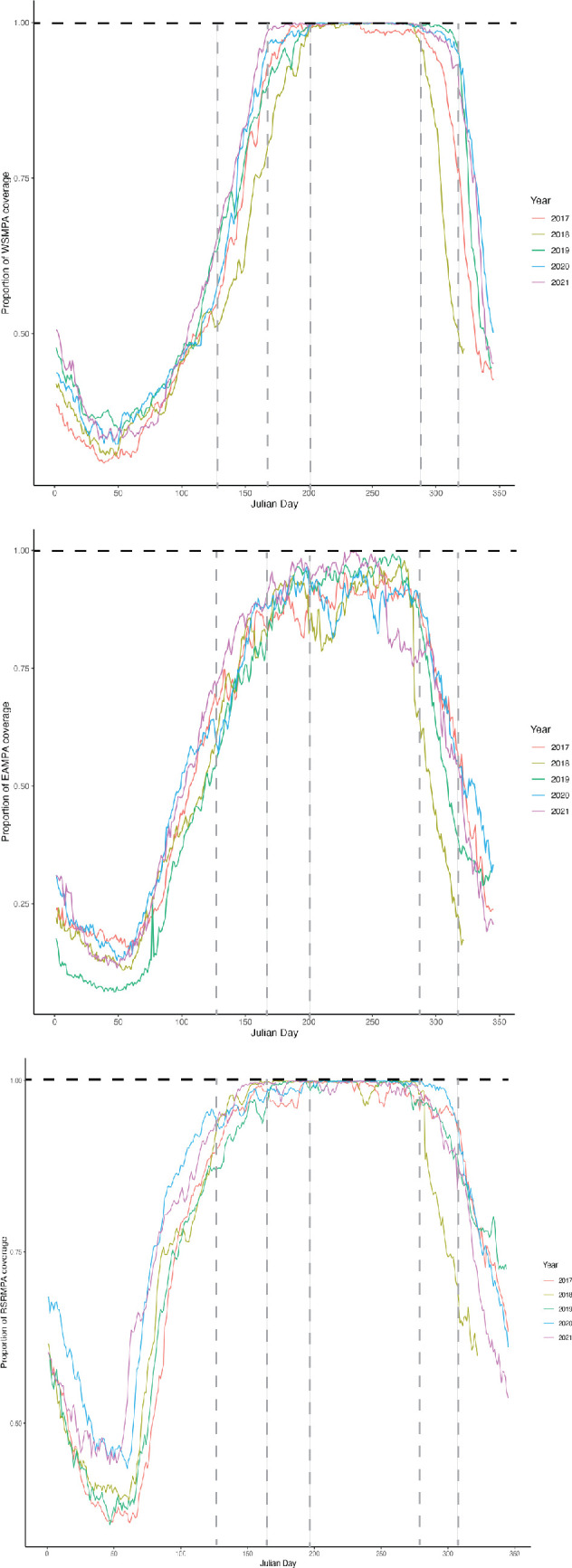
Proportion of coverage by winter sea ice of the Weddell Sea MPA (WSMPA, top panel), East Antarctic MPA (EAMPA, middle panel) and Ross Sea Region MPA (RSRMPA, bottom panel) between 2017 and 2021. Vertical dashed lines represent (left to right) the midpoint of May, June, July, October and November, respectively. The extent of the marginal ice zone during winter fell outside the coverage of the WSMPA and RSRMPA boundaries between mid-May and mid-June. Given the movement patterns of instrumented juvenile emperor penguins from Atka Bay, they would have been excluded from the WSMPA for just over four months after turning south from their initial northward trajectory.

Of the three MPA regions considered in our study, between 2017 and 2021, only the proposed EAMPA would have provided a MIZ year-round (figure 3). The RSRMPA and WSMPA were almost completely ice covered by mid-May and late June, respectively, and remained so until early November (figure 3). Based on telemetry data from birds in this study, fledglings would have been excluded from the WSMPA after approximately one month of returning from their initial foray to the north and would have remained outside its proposed boundaries for just over four months.

## 4. Discussion

Our study supports the pronounced movement patterns shown elsewhere [2–4], in that juvenile emperor penguins performed a long migration to the vicinity of the southern boundary of the Antarctic Circumpolar Current at the northern extents of CCAMLR subareas 48.4 and 48.6 shortly after fledging from their natal colony at Atka Bay (figure 1). Immature king penguins (*Aptenodytes patagonicus*) also used this region, so it may be speculated that it provides good feeding opportunities [2]. Individuals did not remain long at their northern positions, before commencing a rapid southerly movement to within a few hundred km of the MIZ, a pattern remarkably consistent with juvenile emperor penguins tracked from Point Géologie in East Antarctica [3]. The initial migratory movement was broadly synchronous among individuals. The southward movement and subsequent change to area-restricted searching were consistent with the MIZ representing potentially important feeding habitat for the penguins, for example through possibly leading to prey aggregations [30].

The MIZ has long been recognized as an important ecotone for forage species. It provides suitable habitat for their feeding, growth and maturity and resultant high levels of their abundances [30–33]. The structure of the upper ocean, specifically the depth of the upper mixed layer, represents an aggregating boundary of biological productivity against which air-breathing predators forage [34]. Within the MIZ, sub-mesoscale processes at spatiotemporal scales of a few kilometres and hours can have significant impacts on the depth of this boundary, leading to shoaling, particularly in winter [35]. The study at Pointe Géologie showed that within the MIZ juvenile emperor penguins exploited this boundary, presumably feeding on Antarctic krill and mesopelagic fishes that undergo vertical diel migration [4]. Towards the north of their migration, juvenile emperor penguins from Atka Bay also spent a greater proportion of their time at deeper depths. While we acknowledge that prey items may distribute differently within the water column in different regions, it is plausible that the MIZ represents important foraging habitat for young-of-the-year emperor penguins across the species' range.

The diet of emperor penguins comprises a variety of fishes, cephalopods and krill, with Antarctic silverfish *Pleuragramma antarctica* and Antarctic krill *Euphausia superba* among their main prey items [36]. However, the composition of their diet varies between year, colony, season and key foraging area. For example, at Drescher Inlet (72°49′ S, 19°13′ W), in some years the summer diet of adults was dominated by squid species (*Alluroteuthis antarcticus, Moroteuthopsis longimana* and *Psychroteuthis glacialis*, approx. 93% by mass) [37], but on other occasions by Antarctic krill (approx. 75% by mass) [38]. By contrast, emperor penguins at Amanda Bay (69°17’ S, 76°46’ E) mainly consumed Antarctic silverfish (approx. 78%), some squid species and amphipods, but hardly any euphausiids [37–41]. Most prey species of emperor penguins are pelagic, but demersal and benthopelagic species are also consumed [7].

Thus far, our results supported the movement and habitat preferences of juvenile emperor penguins described in other studies. However, in the context of potential threats, their mitigation and science-based advice for managers, we offer an alternative perspective to that presented by [2], which advocated for MPAs that would protect emperor penguins across the ranges of all age categories. In the remainder of the discussion, we elucidate these threats as well as the potential efficacy of suggested management measures.

### 4.1. Potential for trophic competition

In this section, we summarize the potential for emperor penguins of competition with fisheries for food, with emphasis on penguins from Atka Bay. Fisheries that may compete with emperor penguins for food would be those that target krill, cephalopods or fishes in regions used by emperor penguins. However, fisheries for several cephalopods and fish species in the CCAMLR region have not yet proved viable. Within the CCAMLR Convention Area, the contemporary fishery for Antarctic krill is constrained to four discrete areas in the southwest Atlantic (48.1, 2, 3 and 4) and two in the Indian Ocean (subareas 54.8.1 and 2) via a series of management tools codified as CMs. CM 51-01 sets the fishable quota for krill in the southwest Atlantic at 620 000 tonnes per annum, or approximately 1% of the estimated standing stock of area 48 [42]. However, at the time of writing, subarea 48.4 was almost entirely covered by the South Georgia and South Sandwich Islands MPA rendering it effectively closed in terms of economically viable fishing. In the other Atlantic subareas, fishing occurs in a seasonal fashion, with the fishery moving from 48.2 at the end of summer into 48.1, thence into 48.3 (South Georgia) at the onset of winter. Subarea 48.6 currently has no trawl fishing activity; this would be unlikely to change, given the placement of the proposed WSMPA and the winter sea ice extent limiting shelf fishing. Thus, the fishery for krill in the southwest Atlantic, in terms of its spatiotemporal footprint and quota relative to standing stock, is unlikely to pose a direct threat to emperor penguins fledging from Atka Bay, although it may overlap with foraging areas of adults, or of penguins migrating north from more western colonies.

### 4.2. Efficacy of current and proposed spatial management initiatives to protect juvenile emperor penguins

The northern boundary of the CCAMLR Convention Area ranges from 60° S to 45° S. Further north are international high seas, some of which fall within Regional Fisheries Management Organizations, or waters that fall under national jurisdictions. The current and proposed ranges of MPAs within the CCAMLR Convention Area do not extend north of 60° S, the northern boundary of the Antarctic Treaty System. Assuming the MIZ is important to juvenile emperor penguins across their range [3], this feature fell outside the boundaries of existing or proposed spatial management measures around the Southern Ocean for approximately 4.5 months each winter from 2017 to 2022 (figure 3). This creates the possibility that young penguins could compete with fisheries for food outside the MPAs. However, if the effects of climate change continue unabated and sea ice extents reduce, then the current suite of MPAs offers the potential for future protection as the overwinter MIZ recedes southwards.

This leads to an intriguing conundrum. Assuming that the MIZ represents critical feeding habitat that falls exclusively within CCAMLR’s Convention Area, yet outside any spatial-management mechanisms currently under consideration, what science-based advice for managers is appropriate to reduce direct threats that occur in a defined temporal range but across a spatially dynamic habitat feature? Increasing the spatial coverage of existing and planned static-management options to such an extent that they would encompass the MIZ year-round is likely to be politically intractable and has been recognized as inappropriate for far-ranging pelagic marine predators such as emperor penguins [43]. However, dynamic area-based management strategies are becoming more frequently considered as alternative fisheries management tools for protecting non-target pelagic species [44].

The locations of emperor penguin breeding colonies are reasonably well known [15], allowing for modelled estimation of the likelihood of animal movement intersecting with well-monitored fishing efforts. Moreover, the MIZ can be monitored remotely in near-real time. Consequently, given increasing awareness that the MIZ represents important habitat for juvenile emperor penguins, levels of risk to emperor penguin colonies of fishing in proximity to the MIZ could be quantified. This may prove a tractable way to balance increased protection of penguins while minimizing the reduction of exploitable areas for fisheries, e.g. [44,45]. Another option may be to constrain any fishery from operating within a set distance of the MIZ, negating the requirement for year-round, static, spatial-management measures. At present, this is likely to be a self-selected behaviour by the fishery, based on operational safety and the risk of losing fishing gear owing to sea ice, but future technological advances may reduce risks of gear loss.

Given an age at first breeding of 3−8 years and high adult survival (*ca* 95%) for emperor penguins [36], their colonies should be well buffered against recruitment variability. At present the most important threat to emperor penguins remains the potential loss of fast-ice breeding habitat, which is expected to result from climate warming and could lead to substantial losses of eggs and chicks [5]. It will be necessary to halt climate warming to avert this threat, which will require the cooperation of the global community. Spatial management in the CCAMLR Convention Area that maintains healthy levels of juvenile survival will help counter such negative impacts. Greater sample sizes than we provide on the at-sea distributions of young birds will be needed to inform such management. Additionally, substantive information collected on other marine predators in the Southern Ocean can be used to guide spatial-management initiatives (e.g. [46]).

## 5. Conclusion

Declines in sea ice around Antarctica, caused by ongoing climate changes, are likely to drive the MIZ inside the spatial domains of current and proposed MPAs and adjacent regions in the CCAMLR Convention Area. If the MIZ is an important foraging habitat for juvenile emperor penguins, as their altered behaviour on approaching it suggests, achieving the goal of CCAMLR to enact a representative network of MPAs around the continent may benefit emperor penguins in the long term. In terms of contemporary protection, while currently there appear to be limited direct threats posed by human activities such as fishing and tourism, there are proactive steps involving dynamic, spatial management of important pelagic habitat, which CCAMLR could consider as a precautionary approach for the conservation of emperor penguins, particularly given the susceptibility of breeding colonies to global warming. Their implementation may help offset current adverse effects of global warming on breeding by the species while negotiations for MPAs continue and will also set CCAMLR at the forefront of dynamic marine, spatial management. Importantly, as the MIZ is exclusively within the Convention Area, CCAMLR is the only management body that can take such action. However, the present small sample sizes on distributions of immature birds at sea will need to be improved to better inform such measures.

## Data Availability

The dataset generated and analysed during this study is available in the Dryad Data Repository [47].
